# Healthcare providers’ perception about challenges of reproductive health service utilisation

**DOI:** 10.4102/jphia.v16i1.1358

**Published:** 2025-10-28

**Authors:** Meaza G. Sileshi, Lebitsi M. Modiba, Portia J. Jordan

**Affiliations:** 1 Department of Nursing and Midwifery, Faculty of Medicine and Health Sciences, Stellenbosch University, Cape Town, South Africa; 2 Department of Health Studies, Faculty of Human Science, University of South Africa, Pretoria, South Africa

**Keywords:** care providers, challenges, perception, reproductive health, service utilisation

## Abstract

**Background:**

Reproductive health service maintains optimal health and reduces missed opportunities for seropositive women. But it is reported that the service uptake is not adequately recognised.

**Aim:**

This study helped to explore healthcare providers’ perceived challenges of reproductive health service utilisation by seropositive women.

**Setting:**

This study was conducted in Addis Ababa, June 2021.

**Methods:**

Qualitative-exploratory research design was carried out. Human immunodeficiency virus (HIV) programme representative health professionals (*n* = 22) were recruited through the snowball sampling method from different levels of facility and health offices. They participated in three researcher-led focused group discussion (FGD) using semi-structured guiding questions. Prior to actual FGD sessions, a pilot test was done. ATLAS.ti version 7.0 software was used for data analysis. Data visualisation, coding and thematic analysis were done consecutively.

**Result:**

Participants’ mean age was 30.14 years; 12 (54.5%) of them were nurses, and 11 (50%) of them worked for 2–4 years. Low reproductive healthcare utilisation was related to the vertical care delivery approach to HIV, lack of clear guidance for providing multidimensional care, technical skill gaps and the low caring attitude of health professionals. Another factor that lessens reproductive health use was seropositive women’s fear of status disclosure, interest for only HIV care or demanding additional incentives for newly introduced care.

**Conclusion:**

Reproductive health service is inadequately utilised by seropositive women due to several reasons and causes inefficient opportunities to care.

**Contribution:**

This study helps for task-shifting and to design reproductive health components integration with HIV care that ensures comprehensive service to these target population.

## Introduction

Globally, 44% of new human immunodeficiency virus (HIV) infections were reported among women, and 77% of these women were in the reproductive age group and from sub-Saharan Africa.^1^ Reproductive health services emphasise enhancing safe motherhood initiatives and tackling ill maternal health problems and unmet needs for family planning.^2^

Seropositive women have unmet needs towards reproductive well-being for screening and treatment of reproductive tract disorders and contraception method alternatives for spacing their pregnancies.^3^ They also need to have a compassionate and women-friendly approach at the clinic visit, and it is mandatory to give women education and empowerment for improving their current situation.^4,5^

According to assorted studies, there was a tendency of a low rate of sexual and reproductive health component utilisation among HIV-positive clients.^6^ This was mentioned because of healthcare providers’ knowledge, negative attitudes towards professional care, clients’ long waiting hours at health facilities and fear of an attached fee for the services.^7^ Also, despite the availability of services, many women’s adherence was affected by HIV-attached stigma,^8^ inadequate knowledge, lack of service sustainability and lack of appropriate support for the intended medical care. It was mentioned that healthcare providers know the extent of how reproductive healthcare is demanded, and it is obligatory to provide comprehensive care that fills the service gap.^6,9,10,11,12,13^

Reproductive health and HIV service integration policies and guidelines were developed at the national ministry of health level. Moreover, for nearly two decades, the global fund granted a significant amount to carry out such service integration in the country.^14^ However, the pattern of service integration as well as the rate of reproductive healthcare utilisation among seropositive women was not well investigated.

Therefore, reproductive health services could be coordinated with HIV care, mainly with Prevention of Mother-to-Child Transmission (PMTCT) services, to facilitate the journey of safe motherhood. It also helps to address the potential missed opportunities of service integration so that sustainable development goals (SDGs) can be achieved.

### Aim of the study

The aim of this qualitative study was to explore the perception of healthcare providers about challenges of seropositive women in utilising reproductive health services in Ethiopia.

## Research methods and design

### Study setting

This study was carried out in the city of Addis Ababa, Ethiopia. The Addis Ababa regional health sector has 11 big administrative sub-city health bureaus, and under each sub-city, there is a lower level of woreda (district) health offices. Woreda Health Offices are the smallest administrative divisions, equivalent to district level, that control the activity of health centres and community-based health extension programmes.

### Study design

This study utilised an exploratory qualitative design to gather information based on respondents’ personal experience about reproductive health service uptake by HIV-positive women.^15^

### Population and sampling methods

For this study, healthcare professionals who were assigned to the PMTCT unit, health centre managers, HIV focal persons working in PMTCT units and HIV programme coordinators at various levels of health offices were the study population. The list of these healthcare providers who worked in their position for more than 1 year was collected from two purposely selected sub-cities. Then, using the non-probability snowball sampling method, a total of 22 (*n* = 22) study participants were selected to participate in three focus group discussion (FGD) sessions.

### Data collection procedures

The FGD was achieved by discussing with study respondents using a pre-prepared non-structured guiding questionnaire. The FGD was led by the principal investigator and in the local Amharic language. The questionnaire was prepared in English and then translated to the local language, Amharic, as the FGD was held in the local language. The FGD guiding questionnaire consists of questions that help to explore information about the available reproductive health practices in the PMTCT unit, the pattern of service utilisation by seropositive women and the potential challenges that might affect HIV-positive women not enrolling in these comprehensive services.

Before the actual FGD took place, a specific hospital meeting room was arranged with permission from the administrator. Then a pilot test was done by recruiting seven health professionals from sub-city, woreda and health centre levels to check the understandability of questions and to test for the convenience of the physical environment to record voice. The information from the pilot test was included as one of the FGD inputs for the final study. Moreover, when these participants recruited, the principal investigator provided an information sheet that explains about the study for each participant, and written consent was collected before proceeding to FGD. These three consecutive FGDs (*n* = 7, 7 and 8 participants per group) lasted 40 min – 50 min, and each FGD had been documented with a portable audio-recorder and handwritten field notes taken by the principal investigator.

### Data analysis

The recorded audio from the FGD sessions was loaded into Atlas.ti software version 7.0, coded and transcribed verbatim. Initially, thematic areas were identified using an iterative process; that is, the principal researcher listened to the audio-recording and referred to notes, understood transcripts and started to develop descriptive codes inductively that attached to groups of similar ideas. Abbreviated Test Language for All Systems text interpretation (ATLAS.ti) software helps to envision different segments of data and link data with specific code that guides the development of themes. Then, researchers decided to categorise valid sub-themes into the bigger thematic umbrella for giving contextual explanation during qualitative narration.

### Ethical considerations

Ethical clearance to conduct the study was obtained from the University of South Africa (UNISA) Higher Degrees Committee (HSHDC/591/2017), and a support letter was obtained from the Unisa-Ethiopia Regional Learning Center (UNISA-ET/KA/ST/29/07-06-17). The ethical clearance letter was presented to the Addis Ababa city administration ethical review committee for approval. After their permission, the letter was submitted to those two sub-city health bureaus in order to recruit FGD participants from woreda (district) health offices and health facilities. Then, prior to the FGD session, an explanation was made to study participants about the aim of the study and how to assure anonymity and confidentiality, and then their written consent was obtained.

## Results

### Background information of study respondents

A total of 22 (*N* = 22) health professionals participated in FGD, and 14 (63.6%) of them were female professionals. The age range was from 24 years to 38 years, and 77% of them were between 25 years and 34 years of age, with an average age of around 30.14 years. Twelve FGD professionals (54.6%) were nurses with a diploma in nursing (*n* = 3, 13.6%), and nine (41.0%) of them had a Bachelor of Science (BSc) degree in nursing, followed by 36.3% (*n* = 8) of public health professionals at the BSc and Master of Science (MSc) degree levels. Around half of them (50%) had 2–4 years of experience in their current role (refer to Table 1).

**TABLE 1:** Socio-demographic characteristics of focus group participant professionals (*N* = 22).

Demographic characteristics	*n*	%
**Gender**		
Male	8	36.4
Female	14	63.6
**Age in years**		
20–24	1	5.0
25–29	9	41.0
30–34	8	36.0
35–38	4	18.0
**Profession of FGD participants**		
Diploma Nursing	3	13.6
BSc in Nursing	9	41.0
BSc in Midwives	2	9.1
BSc in Health Officer	5	22.7
MSc in Public Health	3	13.6
**Work experience on current role in years**		
1–2	3	14.0
2–4	11	50.0
4	8	36.0

*Source:* Sileshi MG, Modiba LM. Development of reproductive health integrated service delivery model for seropositive women in Addis Ababa, Ethiopia [homepage on the Internet]. Unisa Thesis Repository; 2022. [cited 2022 Sep]. Available from: http://hdl.handle.net/10500/29781

Note: Data derived from the qualitative FGD data on Personal communication of primary investigator and put on tables for the purpose of this manuscript. June 2021.

### Thematic analysis of results

Focused group discussion participants discussed their perceptions about the pattern of and difficulties of utilising reproductive health services by seropositive clients. Based on participants’ responses, three thematic areas were identified, but the first two themes were merged as reproductive health components availability and utilisation and challenges of reproductive health service uptake by seropositive women.

### Reproductive health components availability and utilisation

Focused group discussion respondents mentioned that the HIV Testing and Counselling (HTC) service in the antenatal care (ANC) unit helped them to diagnose pregnant women with HIV and linked them to the PMTCT service as soon as possible. They emphasised that PMTCT schemes of care are the most important approach and the main entry point for HIV and other reproductive health services among seropositive women.

Among the FGD participants, one of them mentioned that:

‘… In previous years, in ANC department when pregnant women were seropositive counsellor link women to ART clinic for WHO staging and initiation of ART or ARV prophylaxis … whereas the current approach allows to have all services and ART follow up in a single care delivery unit, which is PMTCT unit …’ (Participant 19, 29 years, Female, FGD-3)

Discussion participants revealed that some of the health-related components provided in the PMTCT units were ANC care, screening and treatment of sexually transmitted illnesses (STIs) and other reproductive tract illnesses (RTIs). Also, post-natal care (PNC) follow-up, condom use negotiation and distribution, family planning (FP) counselling and methods provision, education on breast self-examination and periodic screening for breast and cervical cancer were mentioned. Although a list of sexual and reproductive health services is available at the health facilities at different levels, few numbers of consumers were reported to uptake those services.

An FGD participant mentioned service availability at the PMTCT unit as:

‘… FP and ANC services are the most demanded services among clients. Whereas services like labour and delivery, postnatal care, STI diagnosis and treatment and emergency contraception have lower rate of service utilization …’ (Participant 12, 33 years, Female, FGD-2)

In general, study participants mentioned that even though reproductive health services help seropositive women in preventive, curative and rehabilitative care, more focus is given to accomplishing HIV components. On the other hand, in spite of the fact that few services were utilised by clients, it is promising to know important components of maternal- and child health-related services are available at health facilities.

### Challenges of reproductive health service uptake

According to the response by FGD participants, reproductive healthcare utilisation among seropositive women was impacted by the approach and quality of service provision. Service provision is directly dependent on resource allocation (guidelines, follow-up, infrastructure, number of staff assigned and availability of time) and providers’ and care seekers’ attitudes, approaches and levels of technical skill. According to this study respondent’s explanation, three categories were explored as possible challenges for seropositive women’s enrolment for reproductive health service uptake related to institutions, clients and care providers.

#### Challenges related to institutions

Focused group discussion respondents expressed their concern that reproductive health services faced a lack of ownership because of their nature not being perceived as independent care by themselves and no service delivery unit named as ‘reproductive health’. Also, the trend of in-service training focused more on traditional and vertical programming styles like family planning, HIV, PMTCT, STI and cervical cancer care than on addressing its holistic components.

One of the FGD participants responded as follows:

‘… I served HIV programs for more than five years, but I do not come across on any guideline stating about integration of reproductive health service with HIV. HIV care is only entertained as the mainstream with independent indicators than developing holistic monitoring tool. So, it is not encouraging to focus on sexual and reproductive health service components other than HIV …’ (Participant 04, 36 years, Male, FGD-1)

Moreover, FGD participants raised issues about the necessity of a holistic care approach for the wellness of seropositive women in the PMTCT clinic. But on the contrary, the actual concern at the care provision spot inclined its effort more to HIV programmes than other reproductive health issues. Mainly peer supporters were hired as lay counsellors to strengthen the vertical approach of HIV care to seropositive clients.

This similar issue was expressed by one of the FGD participants as:

‘… In PMTCT unit, we health care providers and mentor mothers are focusing on ANC follow-up and more on HIV care and treatment. Mostly, other reproductive health issues are not a primary concern and not uniformly given for all clients …’ (Participant 03, 28 years, Female, FGD-1)

The biggest challenge mentioned for lower reproductive health service utilisation was because of gaps in resource allocation and programme monitoring indicators for reproductive healthcare. Human immunodeficiency virus-related services are believed to be more resource-intensive activities and funding agencies are devoted to fulfilling their standards.

One of the FGD participants verbalised this as:

‘… HIV programs are primarily led as projects covered by non-governmental organizations [*NGOs*] with budget for overall cost of the program. … Therefore, care providers might face difficulty to provide comprehensive care …’ (Participant 09, 37 years, Male, FGD-2)

Also, another group discussant clearly mentioned as:

‘… Although health professionals are eager to give all rounded services for their clients, it is impossible to do so due to scarcity of resources, owing to government commitment and poor allocation of budget …’ (Participant 02, 31 years, Male, FGD-1)

On the other hand, even though some care providers were enthusiastic to deliver comprehensive care to seropositive women, they have to spend more time on a single client. This might bring discomfort for dozens of women waiting outside the clinic, and care providers might be criticised for creating longer waiting times. This is created because of the failure of the health system to balance the proportion of client flow to health workers assigned in the specific service delivery units of health facilities:

The FGD participant verbalised the above-mentioned issue as follows:

‘… Some counsellors in the PMTCT unit are eager to apply their training and skills and want to give available services whenever it is needed. But due to heavy load of client flow, mostly they will turn to be selective and only stuck on routine care provision …’ (Participant 04, 36 years, Male, FGD-1)

#### Challenges related to healthcare providers

According to FGD participants, it was mentioned that proper implementation of reproductive health services lacks compassionate attitudes from healthcare providers. Some of the care providers think that clients should get only HIV-related service at the PMTCT unit. If seropositive women want to have a service other than HIV, they are obliged to get appointments or come back again to be referred or get another specific service.

One of the study participants revealed the above-mentioned statement as:

‘… If I were assigned in PMTCT unit, I was only providing available care at the unit. I might link a post-partum client for FP service; else I was not referring to other services like cervical cancer screening test. If a woman wants that service, she can visit the unit by herself …’ (Participant 15, 32 years, Female, FGD-3)

In addition, FGD participants pointed out that health professionals working at service delivery points may not have the confidence to carry out services on reproductive health components. This might be related to the fact that most of the care providers at the PMTCT unit are HIV care experts and might have practical or technical skill gaps, or there is no clear instruction available that instructs them on how to incorporate different services together. This study’s participants believed that healthcare providers’ approach has a big impression on the quality of reproductive health and HIV care delivery. In PMTCT units, healthcare providers were expected to give HIV- and reproductive health-related services like ANC follow-up, post-natal care, exposed infant care and family planning services. But some of these care providers failed to perform those tasks other than PMTCT; rather, they exhibited a low caring attitude and poor work discipline and job satisfaction because of an unfavourable work environment and discomfort in the administrative system.

#### Challenges related to clients

Client-related challenges towards low service utilisation were explained; for most women, it has cultural contexts for being economically dependent on their male sexual companion or because of male partner objection towards fear of HIV-related stigma. Study participants also discussed their concern that some of the seropositive women were not happy to hear about additional services other than HIV. If the care provider initiated a service other than HIV, some of the clients might expect to get financial, in-kind or other support.

According to FGD respondents, the following quote strengthens the mentioned idea:

‘… Even though most of seropositive women engaged in PMTCT follow-up services regularly, they seek for additional support in relation to material or financial base. Sometimes, they think being HIV-positive and having small child is a means of living that increases their eligibility to gain support from community or other sectors …’ (Participant 06, 24 years, Male, FGD-1)

Another important issue raised by FGD respondents was the issue of HIV status disclosure, which might affect seropositive women’s enrolment in reproductive health service packages. In some health centres, care providers might refer their clients to other corresponding units for screening and management of potential reproductive health illnesses. But some ladies failed to use the intended service to keep their secrecy and do not want to disclose their HIV status to other professionals out of the PMTCT unit.

According to the words of a female FGD participant:

‘… In the health centre I am working, we tried to provide all available care and treatment services to all target women. But when we try to refer and link seropositive women for necessary care, most of the time they are not comfortable. Sometimes these clients might disappear from their regular HIV care and support follow up …’ (Participant 21, 27 years, Female, FGD-3)

In general, in the PMTCT unit, the service enrolment of seropositive women for available services like ANC, post-natal care, family planning and STI screening services was not satisfactory.

## Discussion

The pattern of reproductive health services utilisation was highly influenced by service availability and the effort of health system initiatives.^16^ In this study, it was indicated that healthcare institutions faced difficulty allocating resources. This might be because of a lack of programme ownership by the government, and the primary focus of the implementing partners might be advocating for the vertical approach of HIV programmes only.

This study’s participants revealed that reproductive health services practice among HIV-positive women is still weak, which results in a huge gap in unmet needs. A study announced low family planning service enrolment with a high tendency of unplanned pregnancy among HIV-positive young people.^17^ This similarly is because the vertical programming style has got more value than delivering comprehensive services in the healthcare system.

Ayon et al.’s^2^ and Feyissa et al.’s^18^ studies revealed that longer waiting times, unavailability of the intended services, inaccessible care delivery points and the attached cost for the care were major barriers for low service consumption. Other studies stated that healthcare providers’ commitment is directly affected by lack of time to provide additional services because of being overburdened by visiting clients.^12,19^ In the current study, it was mentioned that the care provider to patient ratio is not proportional and causes a delay in reproductive healthcare provision, and clients are obliged to wait for a service for more time. This can be explained by the widespread imbalance in the number of clients and health worker allocation throughout the country, which has a direct relationship with the quality of care provision.

According to some studies, seropositive women were not using reproductive healthcare because of a lack of client-centred service and insufficient knowledge among care providers. Also, fear of potential adverse effects and lack of societal and family approval to utilise reproductive healthcare were additional reasons mentioned.^11,20,21^ In the current study focusing mostly on HIV care, it brought seropositive women to give less value when care providers initiated to provide reproductive health services. This similarity might be because of proper counselling on the importance of seeking other services and poorly constructed monitoring and other quality assurance systems to trace and follow clients.

Even though there is a desire for attending comprehensive healthcare among seropositive women, there is a gap in the pattern of service uptake. Fear of HIV-related stigma, provider skill shortage, the common trend of the vertical HIV care approach, the high number of clients and lack of provider motivation were some of the mentioned reasons.^7,13,22,23^ In the current study, it was explored that healthcare providers insisted on referring clients to other units or health facilities because of a lack of clear guidance or being demotivated to provide care at their specific unit. The similarity of such inadequate reproductive health services utilisation may be related to the fact that most care providers are not happy with the inconducive work environment and administrative issues.

According to the Sileshi and Modiba^24^ study result, seropositive women exhibited low commitment in utilising available services like condom use and STI screening and treatment along with their PMTCT service attendance. Moreover, 44.6% of them revealed their fertility desire to have a child. Another research in southwest Ethiopia also reported low service uptake (26%) for sexual and reproductive health.^25^ In this FGD, it was mentioned repeatedly that utilisation of reproductive health services by seropositive women was not as intended, and as it is indicated in other studies, the primary focus of service attendance among seropositive women might be their only HIV concern, and less value is given for other aspects of care.

In addition to the above-stated reasons, FGD participants emphasised that vertical programming of HIV care, an ambiguous system of care and a lack of guidelines to perform tasks were potential causes for low reproductive health service utilisation. This result is supported by other studies that reproductive healthcare consumption was significantly connected to the level of care providers’ knowledge, availability of health facilities in the vicinity and the degree of open discussion about reproductive health services with others.^25,26^ This similarity between the current and other studies can be related to commonly existing phenomena among care providers in terms of high staff turnover and lack of client awareness of how and where to seek reproductive healthcare.

In general, it was mentioned that health professionals assigned at the service delivery point might face difficulty applying their technical reproductive health-related skills efficiently. Some of them revealed that limited technical knowledge and their dissatisfaction with administrative issues in their actual work settings impacted the availability of the intended services. This study explored that to tackle the actual blockades for reproductive health service uptake, care providers need to understand their scope in integrating HIV with other healthcare, for example, reproductive health. Also, the focus has been emphasised on maintaining the quality of service and changing the approaches of handling clients compassionately.

### Implication to clinical practice

Comprehensive reproductive health service delivery has paramount importance to maintain the quality of life and well-being for seropositive women. It helps to efficiently use scarce resources at the health facilities by addressing multiple issues of target women in a single clinic visit. Moreover, in a service delivery setting, exploring challenges to reproductive health service utilisation helps to guide health facilities to work on resource allocation and capacity building of health professionals. It can give live experience of integrated service provision in the context of HIV and reproductive health, which further enables the emergence of frameworks for service delivery.

### Limitations of the study

This study involved only a few healthcare providers from selected care provision sites in Addis Ababa city. So, as a limitation, most of these FGD respondents had shared similar work environments, and the overall challenges they faced were comparatively similar.

## Conclusion and recommendations

This study explored reproductive health service utilisation, which was not yet comprehensively addressed among seropositive women in Addis Ababa, Ethiopia. The thematic areas from the FGD helped researchers to understand the details of constraints of reproductive health service utilisation. Some of the major challenges mentioned were related to the traditional vertical service provision approach; for example, one service at a time style, poor resource allocation, defective knowledge and attitude of healthcare providers and poor understanding of seropositive women.

It was recommended that health institutions have to create a strategy of integrating services, avail standardised guidelines, provide updated training and create a favourable workplace for care providers. On the care providers’ side, more emphasis should be given to availing appropriate care to seropositive women at each healthcare visit, as it is the only opportunity to provide comprehensive care and to create service-seeking behaviour for underprivileged women. Therefore, healthcare providers should work on creating awareness of the benefits of attending reproductive healthcare along with their HIV follow-up for seropositive women so that service utilisation patterns for both reproductive health and HIV will progress higher.

## References

[1] UNAIDS. Global HIV & AIDS statistics – Fact sheet [homepage on the Internet]. Geneva: 2023. [cited 2024 Jan 18]. Available from: https://www.unaids.org/en/resources/fact-sheet

[2] Ayon S, Jeneby F, Hamid F, Badhrus A, Abdulrahman T, Mburu G. Developing integrated community-based HIV prevention, harm reduction, and sexual and reproductive health services for women who inject drugs. Reprod Health. 2019;16:59. 10.1186/s12978-019-0711-z 31138238 PMC6538559

[3] Ford N, Geng E, Ellman T, et al. Emerging priorities for HIV service delivery. PLoS Med. 2020;17(2):e1003028. 10.1371/journal.pmed.10-0302-8 32059023 PMC7021280

[4] Birdthistle IJ, Fenty J, Collumbien M, et al. Integration of HIV and reproductive health services in public sector facilities: Analysis of client flow data over time in Kenya. BMJ Global Health. 2018;3(5):e000867. 10.1136/bmjhgh-2018-000867 PMC6144905 30245866

[5] Vizheh M, Muhidin S, Moghadam ZB, Zareiyan A. Women empowerment in reproductive health: A systematic review of measurement properties. BMC Womens Health. 2021;21(1):424. 10.1186/s12905-021-01566-0 34930243 PMC8690621

[6] Jisso M, Feyasa MB, Medhin G, et al. Sexual and reproductive health service utilization of young girls in rural Ethiopia: What are the roles of health extension workers? Community-based cross-sectional study. BMJ Open. 2022;12(9):e056639. 10.1136/bmjopen-2021-056639 PMC9494555 36130743

[7] Ormel H, Oele G, Kok M, et al. Reducing unmet need for contraceptive services among youth in Homabay and Narok counties, Kenya: The role of community health volunteers a qualitative study. BMC Health Serv Res. 2021;21:405. 10.1186/s12913-021-06363-x 33933101 PMC8088547

[8] Heeringa J, Mutti A, Furukawa MF, Lechner A, Maurer KA, Rich E. Horizontal and vertical integration of health care providers: A framework for understanding various provider organizational structures. Int J Integr Care. 2020;20(1):2. https://doi.org.10.5334/ijic.4635 10.5334/ijic.4635 PMC6978994 31997980

[9] Adeleye KK, Akinwaare MO, Adejumo PO. Reproductive plans and utilization of contraceptives among women living with HIV. Int J MCH AIDS. 2019;8(2):120–130. 10.21106/ijma.277 31824750 PMC6895771

[10] Bohren MA, Vazquez Corona M, Odiase OJ, et al. Strategies to reduce stigma and discrimination in sexual and reproductive healthcare settings: A mixed-methods systematic review. PLoS Glob Public Health. 2022;2(6):e0000582. 10.1371/journal.pgph.0000582 36962453 PMC10021469

[11] Ngwira GT, Lulin Z. Factors associated with Post-Natal Care (PNC) services utilization among adolescent mothers in rural Northern Malawi. Am J Public Health Res. 2021;9(1):38–47. 10.12691/ajphr-9-1-5

[12] UNAIDS. Global AIDS update seizing the moment tackling entrenched inequalities to end epidemics [homepage on the Internet]. Geneva; 2020 [cited 2024 Jan 18]. Available from: https://www.unaids.org/en/resources/documents/2020/global-aids-report

[13] UNFPA. Evaluation report of UNFPA support to gender equality and women’s empowerment (2012–2020), New York: UNFPA Evaluation Office, 2021.

[14] The Global Fund. HIV Information note. Allocation period 2023-2025 [homepage on the Internet]. Geneva: The Global Fund Advocates Network (GFAN) publications; c2022 [updated 2022 Dec 05; cited 2024 Jan 04]. Available from: https://www.theglobalfund.org/media/14327/cr_hiv_infonote_en.pdf

[15] Polit DF, Beck CT. Nursing research: Generating and assessing evidence for nursing practice. 11th ed. Philadelphia, PA: Wolters Kluwer Health/Lippincott Williams & Wilkins; 2021.

[16] Nkhoma L, Sitali DC, Zulu JM. Integration of family planning into HIV services: A systematic review. Ann Med. 2022;54(1):393–403. 10.1080/07853890.2021.2020893 35098814 PMC8812772

[17] Mbalinda SN, Kaye DK, Nyashanu M, Kiwanuka N. Using Andersen’s behavioral model of health care utilisation to assess contraceptive use among sexually active perinatally HIV-infected adolescents in Uganda. Int J Reprod Medcine. 2020;20:8016483. 10.1155/2020/8016483 PMC7542496 33062664

[18] Feyissa TR, Harris ML, Forder PM, Loxton D. Contraceptive use among sexually active women living with HIV in western Ethiopia. PLoS One. 2020;15(8):e0237212. 10.1371/journal.pone.0237212 32760140 PMC7410321

[19] Milford C, Beksinska M, Greener R, et al. Fertility desires of PLWHIV: Does the implementation of a sexual and reproductive health and HIV integration model change healthcare providers’ attitudes and clients’ desires? BMC Health Serv Res. 2021;21:509. 10.1186/s12913-021-06487-0 34039312 PMC8157636

[20] Odiachi A, Erekaha S, Cornelius LJ, et al. HIV status disclosure to male partners among rural Nigerian women along the prevention of mother-to-child transmission of HIV cascade: A mixed methods study. Reprod Health. 2018;15(36):474. 10.1186/s12978-018-0474-y PMC5833030 29499704

[21] Thomson KA, Telfer B, Awiti PO, Munge J, Ngunga M, Reid A. Navigating the risks of prevention of mother to child transmission (PMTCT) of HIV services in Kibera, Kenya: Barriers to engaging and remaining in care. PLoS One. 2018;13(1):e0191463. 10.1371/journal.pone.0191463 29364979 PMC5783372

[22] Colombini M, Mayhew SH, Dockerty C. Barriers and facilitators to integrating health service responses to intimate partner violence in low- and middle-income countries: A comparative health systems and service analysis special issue: research on integrating sexual and reproductive health and HIV services: Current status, future challenges. Stud Fam Plann. 2017;48(2):179–200. 10.1111/sifp.12021 28422291 PMC5518204

[23] Marotta C, Lochoro P, Pizzol D, et al. Capacity assessment for provision of quality sexual reproductive health and HIV-integrated services in Karamoja, Uganda. Afr Health Sci. 2020;20(3):1053–1065. 10.4314/ahs.v20i3.8 33402951 PMC7751512

[24] Sileshi MG, Modiba LM. Development of reproductive health integrated service delivery model for seropositive women in Addis Ababa, Ethiopia [homepage on the Internet]. Unisa Thesis Repository; 2022. [cited 2022 Sep 28]. Available from: http://hdl.handle.net/10500/29781

[25] Yonas FB, Chernet AA. Sexual and reproductive health service utilization and associated factors among high school learners in the Dawuro zone, Southwest Ethiopia. Afr J Reprod Health. 2022;26(9):48–54. 10.29063/ajrh2022/v26i9 37585069

[26] Dine RD, Uwamahoro V, Oladapo JO, et al. Assessment of the availability, accessibility, and quality of sexual and reproductive health services for young people in conflict affected zones of Cameroon: A mixed method study. BMC Health Serv Res. 2023;23(1): 1159. 10.1186/s12913-023-10142-1 37884966 PMC10601185

